# Relationship between Cervical Instability in the Course of Rheumatoid Arthritis and Pelvic Parameters of Sagittal Balance

**DOI:** 10.3390/jcm12206507

**Published:** 2023-10-13

**Authors:** Robert Wróblewski, Kamil Koszela, Małgorzata Mańczak, Iwona Sudoł-Szopińska, Robert Gasik

**Affiliations:** 1 Department of Neuroorthopedics and Neurology Clinic and Polyclinic, National Institute of Geriatrics, Rheumatology and Rehabilitation in Warsaw, 1 Spartanska Street, 02-637 Warsaw, Poland; 2 Department of Gerontology, Public Health and Didactics, National Institute of Geriatrics, Rheumatology and Rehabilitation in Warsaw, 1 Spartanska Street, 02-637 Warsaw, Poland; 3 Department of Radiology, National Institute of Geriatrics, Rheumatology and Rehabilitation in Warsaw, 1 Spartanska Street, 02-637 Warsaw, Poland

**Keywords:** RA, sagittal spinal balance, spine instability

## Abstract

Background: The aim of the study is to search for a relationship between cervical instability in the course of rheumatoid arthritis (RA) and pelvic parameters of sagittal balance and lumbar lordosis (LL). Methods: The study included 47 patients with rheumatoid arthritis with instability of the cervical segment, who were referred for a consultation to assess indications for surgical treatment. The patients underwent a radiological functional examination of the cervical region and postural examination of the entire spine. The basic parameters of the lumbar section and pelvis of these patients were compared with the population values. Then, using statistical methods, the correlation between radiological parameters of various types of instability and lumbar lordosis (LL), pelvic parameters (PI, SS, PT), as well as the patient’s age and duration of the disease was assessed. Results: A statistical correlation was found between the instability in the cervical spine C2–C3 level and the value of the PT and PI angles. A statistically significant correlation was discovered between C1–C2 instability and younger patient age. There was no significant difference in the values of the pelvic parameters and lumbar lordosis in patients with rheumatoid arthritis compared to the population values. Conclusions: Preliminary results suggest that there is a relationship between selected pelvic parameters and the development of instability in patients with rheumatoid arthritis. This may be important in planning treatment and assessing disease progression. Further studies on a larger group of patients are needed, as well as studies evaluating the correlation between other sagittal balance parameters and cervical instability in patients with RA.

## 1. Introduction

**Figure 1:**
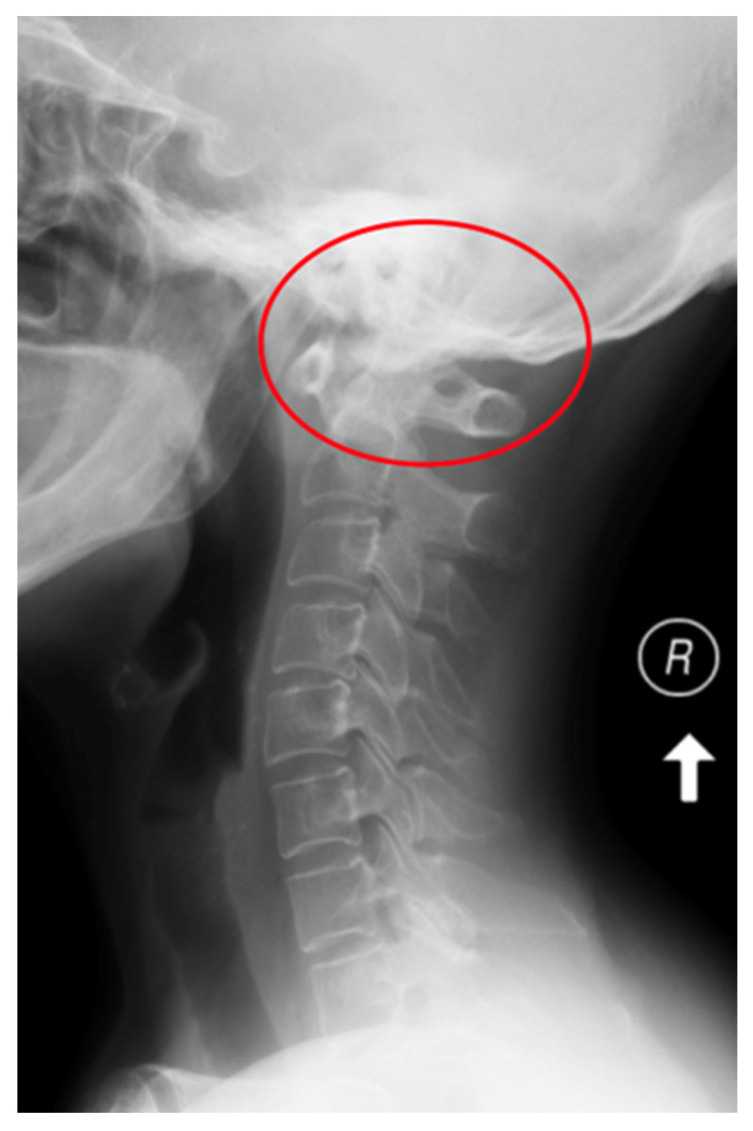
An X-ray of the cervical spine (lateral view) of a 65-year-old female patient with RA and AAS instability.

**Figure 2:**
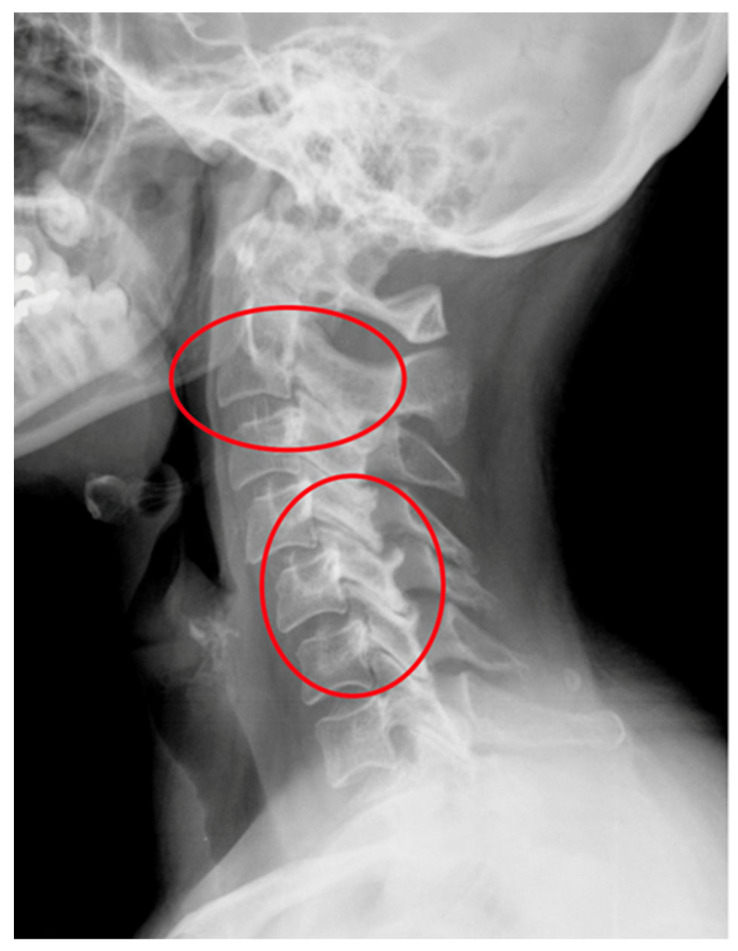
An X-ray of the cervical spine (lateral view) of a 55-year-old female patient with RA and SAS instability.

**Figure 3:**
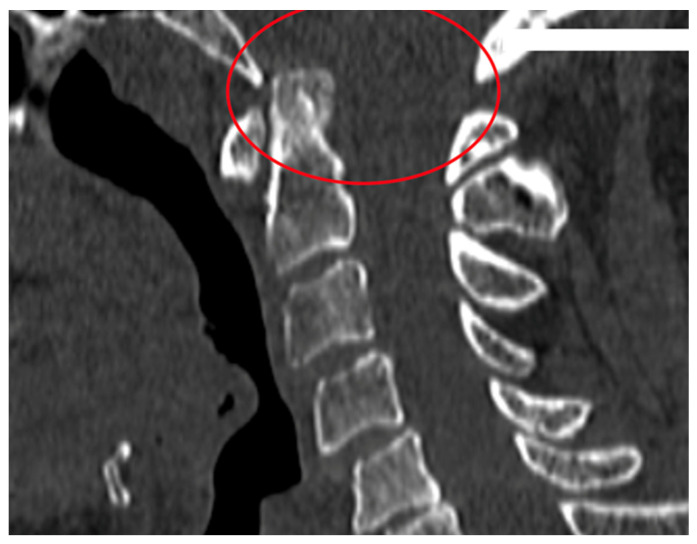
A 61-year-old patient with RA and CrS instability. Cervical CT.

**Figure 4:**
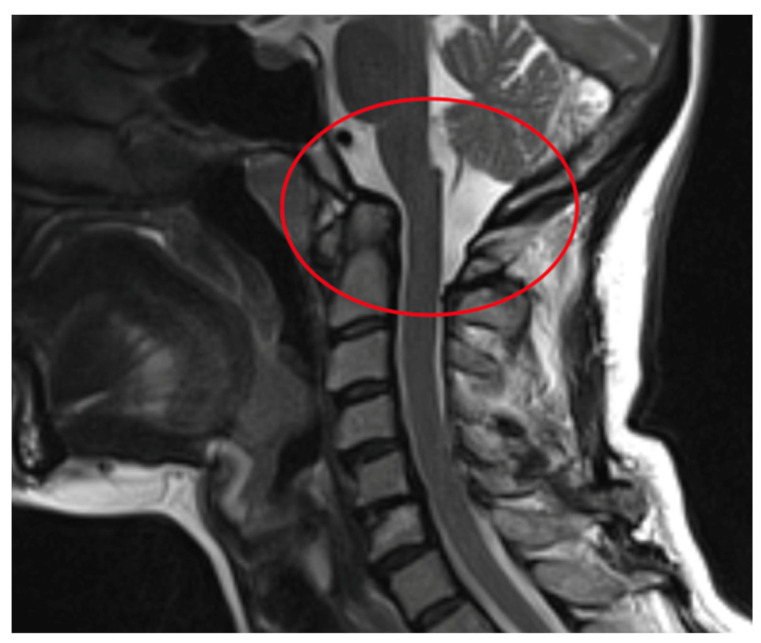
A 61-year-old patient with RA and CrS instability. Cervical MR.

Rheumatoid arthritis (RA) is a chronic inflammatory autoimmune disease [1]. The prevalence of RA in Europe and the USA is estimated at about 0.3–2% of the population over 15 years of age [2]. Factors of an environmental, infectious, genetic and immunological character play a role in the development of RA. The activation of selected genes caused by various extracellular events leads to chronic recurrent inflammation of the synovial membrane and destruction in the joints, and it impacts the cardiovascular, pulmonary, bone and nervous tissues, all leading to systemic complications [1,2]. The inflammatory process in the synovial membrane of the joints leads to joint erosion and damage to ligaments and tendons, creating conditions for mechanical instability [3,4]. The third most common location of the inflammatory process, after the small joints of the hands and feet, are the joints of the cervical spine [3]. One of the consequences of the inflammatory process of the joints is the instability of the cervical spine [4,5]. The cervical segment is the most complex portion of the spine in terms of structure and function. This construction supports the weight of the head, protects the nervous and vascular structures, and at the same time enables the greatest range of motion in relation to other parts of the spine and the compensation of the spatial axial position of the body [6]. Instability of the cervical segment in the course of rheumatoid arthritis may be asymptomatic, but it may manifest itself with severe pain; neurological disorders may occur in 7–35% of patients, and, in extreme cases, this instability may lead to death as a result of compression of the spinal cord and vessels [7,8,9]. There are three types of cervical instability in RA. They are presented in Table 1. They are atlanto-axial subluxation (AAS) (19–70%), which is divided into anterior, lateral and posterior subluxation; then, migration of the odontoid into foramen magnum (cranial settling—CrS) (4–35%), which is divided into lateral dens subluxation (10–20%) and posterior dens subluxation (6–7%); and subaxial subluxation (SAS) (7–29%) [10,11]. Following Figure 1, Figure 2, Figure 3 and Figure 4 are pictures with examples of selected types of cervical instability in RA.

The cause of the disease and effective treatment are not yet known; therefore, it is important to know the factors predisposing one to its development and consequences. Among the factors predisposing one to the development of instability in RA, scientific publications have confirmed female gender, the presence of a rheumatoid factor, prolonged therapy with steroid drugs, early age at the onset of the disease, duration of the disease, underweight, and deforming changes in the joints [12,13,14,15]. The relationship between the occurrence of instability and the presence of antibodies against citrullinated ACPA peptides and metalloproteinase-3 in the serum of patients has also been confirmed [16,17].

Sagittal balance is an issue which, in recent years, has significantly contributed to the expansion of knowledge about the physiology and pathology of the spine [18]. An important matter in the case of patients with rheumatoid arthritis is the generalized nature of the disease that can affect the joints of the entire kinetic chain of the human body. The aim of the study is to check whether pelvic and lumbar parameters are related to the instability of the cervical segment.

## 2. Materials and Methods

This prospective study included patients with RA diagnosed in accordance with the classification criteria for RA developed by ACR and EULAR [19], set in the Clinics of the National Institute of Geriatrics, Rheumatology and Rehabilitation (NIGR & R). The study was approved by the hospital Ethics Committee (No KBT–2/7/2019), and all patients gave their consent. The study involved patients with a long-term course of the disease, on average 21 years (from 2 to 50), who were consulted in the years 2019–2022 at the NIGR & R Clinics in terms of qualification for surgical treatment of instability. Due to the long-term medical history, regardless of the symptoms, the patients had periodic X-ray examinations of the cervical region, in accordance with the recommendations for the care of patients with RA [20,21,22]. The criterium for inclusion in the current study was the diagnosis of AAS or SAS instability in these patients. The definition of AAS instability was an ADI interval (i.e., the distance from the posterior margin of the anterior arch of C1 to the anterior margin of the dens) equal to or greater than 3.0 mm [11]. The definition of SAS instability was a dislocation of the upper end of the posterior wall of the lower vertebra in relation to the lower end of the posterior wall of the upper vertebra equal to or greater than 2 mm [23,24].

Patients meeting the criteria for instability of the cervical region had extended diagnostics with a postural examination of the spine. This examination is dedicated to patients who are considering or planning spinal surgery. The examination was performed on the Carestream DRX Evolution—Health protocol. It was performed in constant conditions (by one radiologist), after assuming a straight, relaxed position with the arms flexed and the fingertips resting on clavicles, while looking ahead [25]. In the radiological assessment of the cervical region, the type and level of instability as well as its degree expressed in millimeters, were taken into account. Pelvic parameters PI, PT and SS as well as the value of lumbar lordosis (LL) were determined in each of the patients in the postural examination. The values of pelvic parameters (PI, PT and SS) and lumbar lordosis (LL) were compared to the normative data of the population [26]. The exclusion criterium was previous surgical treatment of the spine and the inability to perform a postural radiological examination in the patient in a standing position without support.

Definitions of these parameters are presented in Table 2.

The statistical evaluation was performed using IBM SPSS. The distribution of the examined variables was tested using the Kolmogorov–Smirnov tests. The distributions differed from the normal distribution. Non-parametric methods were used for further analyses. Spearman’s correlation coefficients were used to assess the correlations between quantitative variables. Scatter plots were used for significant correlations. Mann–Whitney tests were used to assess the significance of differences between the two groups in terms of quantitative variables. It was assumed that *p* < 0.05 was statistically significant.

## 3. Results

The study involved 47 patients with rheumatoid arthritis, 40 women and 7 men. The mean age of women was 65 years (32 to 89 years); the mean age of men was 62 years (31 to 81 years). In the radiological assessment of the cervical region, the level and degree (in millimeters) of the instability as well as the values of PT, PI and SS angles and LL were taken into account. The correlation of instability with age and disease duration was also investigated. In the study group, C1–C2 instability occurred in 27 patients (32.1%), C2–C3 in 10 patients (11.9%), C3–C4 in 15 patients (17.8%), C4–C5 in 21 patients (25%), C5–C6 in 9 patients (10.7%), C6–C7 in 2 patients (2.3%) and CrS in 3 cases (3.4%). In our calculations, we focused on instabilities in which the dominant component was the force related to the horizontal displacement of the vertebral bodies relative to each other (shear forces), i.e., atlanto-axial subluxation (AAS) and subaxial subluxation (SAS). Spearman correlation coefficients between intervertebral distances in groups of patients with instabilities at different levels are presented in Table 3.

**Figure 5:**
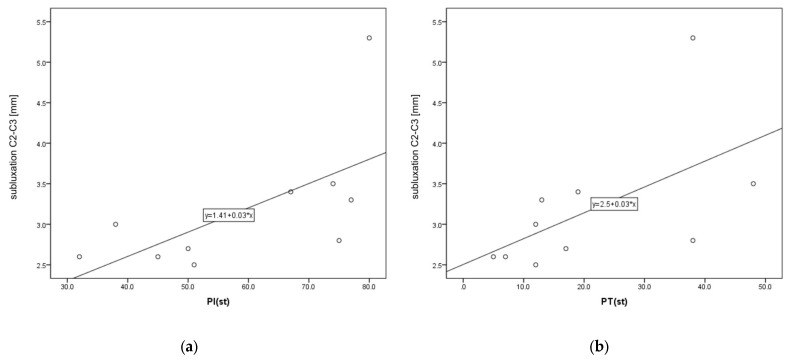
Scatter plots for C2–C3 and PI (**a**) and PT (**b**).

A statistically significant strong positive correlation was found between C2–C3 distance and two sagittal balance parameters: PI and PT (rho = 0.663 and rho = 758, respectively). We also found weak negative correlation between patient age and C1–C2 distance (rho = −0.303). Other values of correlation coefficients between the analyzed parameters were not statistically significant. Scatter plots for C2–C3 and PI and PT are presented in Figure 5.

Patients with AAS were found to be younger than patients without AAS: 64 vs. 71 years (*p* = 0.039). There were no other differences between AAS patients and non-AAS patients (Table 4).

## 4. Discussion

In addition to systemic factors predisposing to the development of cervical instability in RA, examples of which are listed in the introduction, another group of factors that, in recent years, has been attracting the attention of researchers dealing with spinal diseases are biomechanical factors [18]. Along with the advancement of knowledge on the role of sagittal balance in spinal pathology and planning surgical treatment of the spine in various diseases, there appeared studies on the issue of sagittal balance in patients with RA [27,28,29]. Apart from destructive lesions in the joints affecting the mechanics of the cervical spine, it has been observed that changes predisposing to the development of instability are vertebral stiffening resulting either from the natural course of the disease, e.g., ankylosis, or previous surgical treatment of the cervical section, i.e., arthrodesis [30,31,32,33].

In scientific studies regarding other diseases and the aging of the spine compensatory mechanisms of sagittal balance, in which the cervical and pelvis play a key role, have been well described [34,35]. Patients with RA, however, are characterized by differences compared to patients with other diseases of the spine. An important issue in the case of patients with RA is to take into account the generalized nature of the disease affecting the joints of the entire kinetic chain of the human body [36]. Due to joint lesions characteristic of RA, these patients may experience lesions not observed in other diseases or in healthy subjects.

The values of the pelvic parameters in the studied patients compared to the normative population values, unlike the results of the study by Heong Seok Lee et al., did not differ significantly [28]. AAS instability is the most common instability in RA patients described in scientific literature [10,11]. The acquired observations are similar in that respect, i.e., regarding 27 patients (32.1%); however, a statistically significant correlation between AAS instability and LL values and pelvic parameters in these patients has not been found. On the other hand, a correlation between PT and PI values and C2–C3 instability (Table 4) has been found. The obtained result prompted a more detailed look at SAS instability as well as focused attention on the differences in the structure and function of the C2–C3 segment. It also forced a more careful look at the huge role of PT and PI in the sagittal balance of the body.

Among the anatomical and functional factors that may affect the biomechanical properties of the C2–C3 segment, the following ones can be noted:

- At the C2–C3 level, the axial load of the skull and spine is divided.

Loads running from the COG point (center of gravity) through the occipital condyles, C1 lateral masses, C1–2 joints and C2 lateral masses are divided into anterior and posterior columns. The anterior column (passing through the intervertebral disc C2–C3) is one third of the axial load of C3; the posterior column (passing through two symmetrical intervertebral joints C2–C3) is two thirds of the axial load of C3 [37,38].

- At the C2–C3 level, a connection/combination of two functionally and anatomically different parts of the cervical section occurs, i.e., the supraspinatus part, in which the structure, apart from flexion and extension, is subordinated to rotational function and is responsible for 75% of this movement of the occiput in relation to C2, and the subscapularis part, in which flexion, extension and lateral flexion are dominant [6].

This segment is the beginning of the typical cervical segment, with the preservation of differences during the axial rotation of the neck, i.e., with lateral flexion, and the direction of the coupling in C2–C3 is opposite to that observed in the lower parts of the cervical segment [6].

- In a study of the impact of the instantaneous center of rotation (ICR) on the pathology of the cervical spine, it was found that most of the abnormal ICRs associated with neck pain are located at the C2–C3 and C3–C4 levels [39]. However, it has not yet been confirmed that an abnormal ICR is an indicator of damage to this segment. It has been found that it can be used to determine whether it is associated with muscle tone disorders or ligament tone disorders [6]. In both cases, we are dealing with a situation that corresponds to the response to overloading of the spinal structures.

The observations made and the obtained results indicate that further research is needed into the biomechanical properties of this segment and the biomechanical and functional diversity of SAS in patients with RA.

In the case of the relationship between the instability of the C2–C3 segment and the PI and PT values, it is noteworthy that a statistically positive correlation was found between them, i.e., that with the increase of PI and PT values, the degree of instability at this level also increases. The values of SS, PT and PI are interdependent according to the formula PI = PT + SS [40]. The results suggest no correlation between SS and LL and cervical instability. Both of these radiological parameters are of great importance in the vertebral balance of the spine and reflect the importance of the inclination of the S1 endplate to the horizontal line and the relationship of this inclination with the LL angle [41,42].

Therefore, attention should be paid to another component of the equation, the value associated with the increase in the PT and PI angle, i.e., parameters reflecting the biomechanical significance of the distance between the sagittal vertical axes (SVAs), one of which passes through the center of the upper S1 endplate or through the posterior upper endplate, and the other passes through the femoral head center (FH SVA). The distance between these axes is referred to as the sacro-femoral distance (SFD). An increase in the PT and a larger value of the PI angle causes a translational separation of these axes. The found relationship may reflect the nature of the biomechanical relationship between the structure and spatial behavior of the pelvis with the development of the instability of the C2–C3 segment. In a properly balanced posture, the axis drawn from the COG, i.e., CrSVA, heading to the support quadrangle of the feet, passes through the heads of the femoral bones. In turn, the plumbline C7SVA, which is currently the main parameter of sagittal alignment in a balanced posture, is referenced and directed from the S1 endplate posterior corner [43]. In studies on sagittal balance, the parameter described for these variables is the Barrey index [34]. This is a proportion parameter; therefore, it excludes positioning errors. It is the ratio of C7PL (plumbline distance from the S1 endplate posterior corner) to SFD (sacro-femoral distance). The normal mean C7PL/SDF ratio is assumed to be −0.9 ± 1 [34]. However, while focusing on the cervical section of the kinetic chain, in studies on the instability of this cervical section, we should remember that the optimal course of the axis of gravity is the course from the center of gravity of the head (COG SVA), through the center of C2, C7 and the head of the femoral bone, through the knee joints to the area of the base determined by the position of the feet. An explanation of these relationships may have been advanced by the work of Y. C. Kim et al., which showed that CrSVA is a stronger predictor of preoperative clinical outcomes in adult patients than C7 SVA [44]. The correlation between translation of the axes through the COG and CrSVA-S (S1 endplate posterior corner), CrSVA-H (femoral heads), CrSVA-K (knees) and CrSVA-A (ankles) was investigated, and all were found to be related to the quality of life of the patients [44]. Further observations are needed. It seems that obtained results may confirm the importance of research on these variables of human sagittal balance and their impact on the development of instability.

The last observation is the demonstration of a correlation that contradicts intuitive reasoning, i.e., the relationship of instability at the C1–C2 level with the younger age of patients. A number of factors could have influenced this result. One of them is the early start of treatment with DMARDs (disease-modifying anti-rheumatic drugs), which more effectively inhibits joint destruction. In addition, our study involved patients who could assume a free, upright position during the examination, i.e., subjects whose compensatory mechanisms allowed them to maintain alignment even with significant joint destruction. Another observation is that peak incidence of RA occurs in the 4th and 5th decades of life [2]. According to Schwab et al., as many as 68% of people over 60 years of age had ASD (adult spine deformity) [45]. It can be seen that the period in which the decrease in the frequency of diagnosed AAS is observed coincides with the period of development of ASD changes. However, a risky thesis can be put forward that lesions appearing with age in the human balance, described by Roussouly et al. in the study on the aging of the spine, may have a protective effect on the C1–C2 level of the spine, which is most at risk due to its ligamentous and articular structure [46]. Conclusions related to age and RA duration based on our group of patients must be cautious.

When planning the treatment of patients with RA, preliminary observations and results may serve to direct attention to the PT and PI parameters and their probable impact on cervical segment.

The limitations of this study were the number of patients and the lack of consensus regarding the definition of the diagnosis of SAS instability. Some authors diagnose SAS when horizontal displacement of vertebrae is more than 3.5 mm [11]. These issues require further research on a larger group of patients and involving other parameters of sagittal balance.

## 5. Conclusions

1. A statistically significant positive correlation was found between C2–C3 instability and PI and PT. There was no statistical correlation between the pelvic parameters and LL and other levels of instability.
2. A statistically significant negative correlation was observed between C1–C2 instability and the age of patients.

## Figures and Tables

**Table 1:** Types of cervical instability in RA.

Type of Instability	Definition
AAS (atlantoaxial subluxation)	Weakening or rupture of ligaments and subchondral bone erosion in the atlantoaxial C1–C2 joints.
SAS (subaxial subluxation)	Subluxation in the joints C2–C7 due to destruction of the joint surface and the ligaments between the processes spinosis.
CS (cranial settling)	Vertical translocation of dens C0–C2 into the foramen magnum.

**Table 2:** Parameters of sagittal balance.

Parameter	Definition
LL (Lumbar Lordosis)	Measured between the upper endplate L1 and the upper endplate S1.
PI (Pelvic Incidence)	The angle formed between the perpendicular line to the center point of the superior sacrum endplate surface and the line connecting from the said center point to the center of the femur head.
PT (Pelvic Tilt)	This is the angle between the upper plumb line from the femur head center and the center point of the superior sacrum endplate surface.
SS (Sacral Slope)	This is the angle between a horizontal line and the slope of the superior sacral endplate surface.

**Table 3:** Correlations between cervical instability and parameters of sagittal balance, age and disease duration. Statistically significant coefficients at *p* < 0.05 are presented in bold.

	C1–C2 (*n* = 47)	C1–C2 AAS (*n* = 27)	C2–C3 SAS (*n* = 10)	C3–C4 SAS (*n* = 15)	C4–C5 SAS (*n* = 21)	C5–C6 SAS (*n* = 9)
LL(st)	0.041	0.006	−0.322	−0.168	−0.084	0.443
PI(st)	0.077	−0.238	**0.663**	0.320	−0.084	−0.219
PT(st)	0.164	−0.014	**0.758**	0.213	0.132	−0.266
SS(st)	−0.005	−0.316	0.215	0.323	−0.156	0.184
age	**−0.303**	−0.073	−0.261	0.050	0.168	−0.333
disease duration	0.133	0.081	0.500	−0.463	0.315	−0.545

**Table 4:** Comparison of sagittal balance parameters, age and disease duration between patients with and without AAS.

	AAS		Without AAS		*p*
	Me (IQR)	*n*	Me (IQR)	*n*	
LL(st)	54 (30–64)	27	48 (34–56)	20	0.576
PI(st)	53 (44–69)	27	49 (41–56)	19	0.246
PT(st)	16 (12–28)	27	18 (13–20)	19	0.441
SS(st)	36 (27–42)	27	32 (26–38)	20	0.401
age	64 (58–70)	27	71 (63–76)	20	0.039
disease duration	20 (12–30)	26	15 (10–25)	19	0.395

## Data Availability

The data are available from the corresponding author if required.

## References

[1] Smolen J.S., Aletaha D., Barton A., Burmester G.R., Emery P., Firestein G.S., Kavanaugh A., McInnes I.B., Solomon D.H., Strand V. (2018). Rheumatoid arthritis. Nat. Rev. Dis. Primers.

[2] Szczeklik A. (2020). Interna 2020.

[3] Canale S.T., Beaty J.H. (2016). Campbell’s Operative Orthopedics.

[4] Wasserman B.R., Moskovich R., Razi A.E. (2011). Rheumatoid arthritis of the cervical spine—Clinical considerations. Bull. NYU Hosp. Jt. Dis..

[5] Gillick J.L., Wainwright J., Das K. (2015). Rheumatoid arthritis and the cervical spine: A review on the role of surgery. Int. J. Rheumatol..

[6] Bogduk N., Mercer S. (2000). Biomechanics of the cervical spine. I: Normal kinematics. Clin. Biomech..

[7] Nguyen H.V., Ludwig S.C., Silber J., Gelb D.E., Anderson P.A., Frank L., Vaccaro A.R. (2004). Rheumatoid arthritis of the cervical spine. Spine J..

[8] Mathews J.A. (1998). Wasting of the small hand muscles in upper and mid-cervical cord lesions. QJM.

[9] Neva M.H., Myllykangas-Luosujarvi R., Kauppi M. (2001). Mortality associated with cervical spine disorders: A population—Based study of 1666 patients with rheumatoid arthritis who died in Finland in 1989. Rheumatology.

[10] Krauss W.E., Bledsoe J.M., Clarke M.J., Nottmeier E.W., Pichelmann M.A. (2010). Rheumatoid arthritis of the craniovertebral junction. Neurosurgery.

[11] Mańczak M., Gasik R. (2017). Cervical spine instability in the course of rheumatoid arthritis—Imaging methods. Reumatologia.

[12] Zhu S., Xu W., Luo Y., Zhao Y., Liu Y. (2017). Cervical spine involvement risk factors in rheumatoid arthritis: A meta-analysis. Int. J. Rheum. Dis..

[13] Geraldo-Flores N.A., Merlos-López R.J., Rodríguez-Wong J.A., Ramírez-Hernández S., Espino-Lizarraga M.J., Pérez-Atanasio J.M. (2018). La severidad de la artritisreumatoidecomo predictor oportuno de inestabilidad de la columna cervical asintomática [The severity of rheumatoid arthritis as a timely predictor of instability in the asymptomatic cervical spine]. Acta Ortop. Mex..

[14] Yurube T., Sumi M., Nishida K., Miyamoto H., Kohyama K., Matsubara T., Miura Y., Hirata H., Sugiyama D., Doita M. (2014). Accelerated development of cervical spine instabilities in rheumatoid arthritis: A prospective minimum 5-year cohort study. PLoS ONE.

[15] Terashima Y., Yurube T., Hirata H., Sugiyama D., Sumi M., Hyogo Organization of Spinal Disorders (2017). Predictive Risk Factors of Cervical Spine Instabilities in Rheumatoid Arthritis: A Prospective Multicenter Over 10-Year Cohort Study. Spine.

[16] Baek I.W., Joo Y.B., Park K.S., Kim K.J. (2021). Risk factors for cervical spine instability in patients with rheumatoid arthritis. Clin. Rheumatol..

[17] Kaito T., Ohshima S., Fujiwara H., Makino T., Takenaka S., Sakai Y., Yoshikawa H. (2019). Predictors for progression of two different types of cervical lesions in rheumatoid arthritis treated with biologic agents. J. Orthop. Sci..

[18] Dubousset J. (2019). Spinal Alignment, Balance and Harmony through the Ages. Int. J. Orth..

[19] Aletaha D., Neogi T., Silman A.J., Funovits J., Felson D.T., Bingham C.O., Birnbaum N.S., Burmester G.R., Bykerk V.P., Cohen M.D. (2010). 2010 rheumatoid arthritis classification criteria: An American College of Rheumatology/European League Against Rheumatism collaborative initiative. Ann. Rheum. Dis..

[20] Colebatch A.N., Edwards C.J., Østergaard M., van der Heijde D., Balint P.V., D’Agostino M.-A., Forslind K., Grassi W., Haavardsholm E.A., Haugeberg G. (2013). EULAR recommendations for the use of imaging of the joints in the clinical management of rheumatoid arthritis. Ann. Rheum. Dis..

[21] Joaquim A.F., Ghizoni E., Tedeschi H., Appenzeller S., Riew K.D. (2015). Radiological evaluation of cervical spine involvement in rheumatoid arthritis. Neurosurg. Focus.

[22] Siempis T., Tsakiris C., Anastasia Z., Alexiou G.A., Voulgaris S., Argyropoulou M.I. (2023). Radiological assessment and surgical management of cervical spine involvement in patients with rheumatoid arthritis. Rheumatol. Int..

[23] Yurube T., Sumi M., Nishida K., Miyamoto H., Kohyama K., Matsubara T., Miura Y., Sugiyama D., Doita M., Kobe Spine Conference (2012). Incidence and aggravation of cervical spine instabilities in rheumatoid arthritis: A prospective minimum 5-year follow-up study of patients initially without cervical involvement. Spine.

[24] Yonezawa T., Tsuji H., Matsui H., Hirano N. (1995). Subaxial lesions in rheumatoid arthritis. Radiographic factors suggestive of lower cervical myelopathy. Spine.

[25] Faro F.D., Marks M.C., Pawelek J., Newton P.O. (2004). Evaluation of a functional position for lateral radiograph acquisition in adolescent idiopathic scoliosis. Spine.

[26] Cirillo Totera J.I., Fleiderman Valenzuela J.G., Garrido Arancibia J.A., Pantoja Contreras S.T., Beaulieu Lalanne L., Alvarez-Lemos F.L. (2021). Sagittal balance: From theory to clinical practice. EFORT Open Rev..

[27] Masamoto K., Otsuki B., Fujibayashi S., Shima K., Ito H., Furu M., Hashimoto M., Tanaka M., Lyman S., Yoshitomi H. (2018). Factors influencing spinal sagittal balance, bone mineral density, and Oswestry Disability Index outcome measures in patients with rheumatoid arthritis. Eur. Spine J..

[28] Lee H.S., Lee J.S., Shin J.K., Goh T.S. (2017). Correlations Between Sagittal Spinal Balance and Quality of Life in Rheumatoid Arthritis. Clin. Spine Surg..

[29] Ha B.J., Won Y.D., Ryu J.I., Han M.H., Cheong J.H., Kim J.M., Chun H.J., Bak K.H., Bae I.S. (2020). Relationship between the atlantodental interval and T1 slope after atlantoaxial fusion in patients with rheumatoid arthritis. BMC Surg..

[30] Iizuka H., Iizuka Y., Okamura K., Yonemoto Y., Mieda T., Takagishi K. (2017). Bony ankylosis of the facet joint of the cervical spine in rheumatoid arthritis: Its characteristics and relationship to the clinical findings. Mod. Rheumatol..

[31] Ito H., Neo M., Sakamoto T., Fujibayashi S., Yoshitomi H., Nakamura T. (2009). Subaxial subluxation after atlantoaxial transarticular screw fixation in rheumatoid patients. Eur. Spine J..

[32] Wu X., Qi Y., Tan M., Yi P., Yang F., Tang X., Hao Q. (2018). Incidence and risk factors for adjacent segment degeneration following occipitoaxial fusion for atlantoaxial instability in non-rheumatoid arthritis. Arch. Orthop. Trauma Surg..

[33] Kurogochi D., Takahashi J., Uehara M., Ikegami S., Kuraishi S., Futatsugi T., Oba H., Takizawa T., Munakata R., Hatakenaka T. (2019). Ten-Year Results of Reconstruction for Rheumatoid Cervical Spine Lesions and Occurrence Factor of Subaxial Subluxation. Asian Spine J..

[34] Barrey C., Roussouly P., Le Huec J.C., D’Acunzi G., Perrin G. (2013). Compensatory mechanisms contributing to keep the sagittal balance of the spine. Eur. Spine J..

[35] Roussouly P., Pinheiro-Franco J., Labelle H., Gechren M. (2019). Sagittal Balance of the Spine: From Normal to Pathology: A Key for Treatment Strategy.

[36] Błaszczyk J. (2004). Biomechanikakliniczna, Podręcznikdlastudentówmedycynyifizjoterapii.

[37] Scheer J.K., Tang J.A., Smith J.S., Acosta F.L., Protopsaltis T.S., Blondel B., Bess S., Shaffrey C.I., Deviren V., Lafage V. (2013). Cervical spine alignment, sagittal deformity, and clinical implications: A review. J. Neurosurg. Spine.

[38] Louis R. (1985). Spinal stability as defined by the three-column spine concept. Anat. Clin..

[39] Amevo B., Aprill C., Bogduk N. (1992). Abnormal instantaneous axes of rotation in patients with neck pain. Spine.

[40] Duval-Beaupère G., Schmidt C., Cosson P. (1992). A Barycentremetric study of the sagittal shape of spine and pelvis: The conditions required for an economic standing position. Ann. Biomed. Eng..

[41] During J., Goudfrooij H., Keessen W., Beeker T.W., Crowe A. (1985). Toward standards for posture. Postural characteristics of the lower back system in normal and pathologic conditions. Spine.

[42] Legaye J., Duval-Beaupère G., Hecquet J., Marty C. (1998). Pelvic incidence: A fundamental pelvic parameter for three-dimensional regulation of spinal sagittal curves. Eur. Spine J..

[43] Jackson R.P., McManus A.C. (1994). Radiographic analysis of sagittal plane alignment and balance in standing volunteers and patients with low back pain matched for age, sex, and size. A prospective controlled clinical study. Spine.

[44] Kim Y.C., Lenke L.G., Lee S.J., Gum J.L., Wilartratsami S., Blanke K.M. (2017). The cranial sagittal vertical axis (CrSVA) is a better radiographic measure to predict clinical outcomes in adult spinal deformity surgery than the C7 SVA: A monocentric study. Eur. Spine J..

[45] Schwab F., Lafage V., Boyce R., Skalli W., Farcy J.P. (2006). Gravity line analysis in adult volunteers: Age-related correlation with spinal parameters, pelvic parameters, and foot position. Spine.

[46] Roussouly P., Pinheiro-Franco J.L. (2011). Biomechanical analysis of the spino-pelvic organization and adaptation in pathology. Eur. Spine J..

